# Advanced one-pot deconstruction and valorization of lignocellulosic biomass into triacetic acid lactone using *Rhodosporidium toruloides*

**DOI:** 10.1186/s12934-022-01977-0

**Published:** 2022-12-08

**Authors:** Peter B. Otoupal, Gina M. Geiselman, Asun M. Oka, Carolina A. Barcelos, Hemant Choudhary, Duy Dinh, Wenqing Zhong, HeeJin Hwang, Jay D. Keasling, Aindrila Mukhopadhyay, Eric Sundstrom, Robert W. Haushalter, Ning Sun, Blake A. Simmons, John M. Gladden

**Affiliations:** 1 Biomanufacturing and Biomaterials Department, Sandia National Laboratories, Livermore, CA USA; 2 DOE Joint BioEnergy Institute, Lawrence Berkeley National Laboratory, Emeryville, CA USA; 3 Agile BioFoundry, Department of Energy, Emeryville, CA USA; 4 Advanced Biofuels and Bioproducts Process Development Unit, Lawrence Berkeley National Laboratory, Emeryville, CA USA; 5 Biological Systems and Engineering, Lawrence Berkeley National Laboratory, Berkeley, CA USA; 6 Department of Chemical & Biomolecular Engineering, University of California, Berkeley, Berkeley, CA USA; 7 Department of Bioengineering, University of California, Berkeley, Berkeley, CA USA; 8 Center for Biosustainability, Danish Technical University, Lyngby, Denmark; 9 Center for Synthetic Biochemistry, Institute for Synthetic Biology, Shenzhen Institute of Advanced Technology, Shenzhen, China; 10 Environmental Genomics and Systems Biology Division, Lawrence Berkeley National Laboratory, Berkeley, CA USA

## Abstract

**Background:**

*Rhodosporidium toruloides* is capable of co-utilization of complex carbon sources and robust growth from lignocellulosic hydrolysates. This oleaginous yeast is therefore an attractive host for heterologous production of valuable bioproducts at high titers from low-cost, deconstructed biomass in an economically and environmentally sustainable manner. Here we demonstrate this by engineering *R. toruloides* to produce the polyketide triacetic acid lactone (TAL) directly from unfiltered hydrolysate deconstructed from biomass with minimal unit process operations.

**Results:**

Introduction of the 2-pyrone synthase gene into *R. toruloides* enabled the organism to produce 2.4 g/L TAL from simple media or 2.0 g/L from hydrolysate produced from sorghum biomass. Both of these titers are on par with titers from other better-studied microbial hosts after they had been heavily engineered. We next demonstrate that filtered hydrolysates produced from ensiled sorghum are superior to those derived from dried sorghum for TAL production, likely due to the substantial organic acids produced during ensiling. We also demonstrate that the organic acids found in ensiled biomass can be used for direct synthesis of ionic liquids within the biomass pretreatment process, enabling consolidation of unit operations of in-situ ionic liquid synthesis, pretreatment, saccharification, and fermentation into a one-pot, separations-free process. Finally, we demonstrate this consolidation in a 2 L bioreactor using unfiltered hydrolysate, producing 3.9 g/L TAL.

**Conclusion:**

Many steps involved in deconstructing biomass into fermentable substrate can be combined into a distinct operation, and directly fed to cultures of engineered *R. toruloides* cultures for subsequent valorization into gram per liter titers of TAL in a cost-effective manner.

**Supplementary Information:**

The online version contains supplementary material available at 10.1186/s12934-022-01977-0.

## Background

Environmental and health concerns related to the manufacturing and use of petroleum-derived fuels and chemicals have sparked a demand for environmentally sustainable alternatives. Secondary metabolites such as polyketides and non-ribosomal peptides have generated significant interest because of their potential use in a wide range of industries [1]. Triacetic acid lactone (TAL; 4-hydroxy-6-methyl-2-pyrone) is an example of a polyketide that has attracted interest as a promising platform biomolecule [2, 3]. TAL is capable of undergoing chemical conversion to sorbic acid, fungicides, and valuable chemicals such as resorcinol, phloroglucinol, and 1,3,5-trihydroxybenezene [4]. Other applications include polymers, plasticizers, organic synthesis, adhesives and emulsifiers [5]. While it has industrial potential as a sustainable source of green chemicals, TAL production is currently limited by the usage of petroleum feedstock, ecologically unfriendly catalysis, and the formation of toxic byproducts [6].

Consequently, there is a compelling need to build a low-cost, environmentally-friendly platform for TAL production from renewable feedstocks. Great progress has been made over the last decade in engineering biological routes towards TAL production, particularly in yeasts. Much of this work has focused on engineering the canonical yeast, *Saccharomyces cerevisiae*, to produce TAL. While initial titers were low (56 mg/L) [7], significant metabolic engineering efforts [8, 9] have dramatically increased titers of TAL from *S. cerevisiae* nearly 100-fold, up to 5.2 g/L [10]. Consistently greater titers have since been achieved with the oleaginous ascomycete, *Yarrowia lipolytica* [5, 11], where researchers have produced up to 36 g/L [12].

Another oleaginous yeast, the basidiomycete *Rhodosporidium toruloides* (also known as *Rhodotorula toruloides*), is a promising host for the conversion of lignocellulosic biomass into bioproducts [13–15]. *R. toruloides* cultures are capable of surpassing cell densities of 150 g/L dry cell weight [16], are resistant to strong osmotic stress [17], are not hindered by the presence of (potential) growth inhibitors that are typically found in lignocellulosic hydrolysates [18] and adapt well to co-utilization of mixed carbon sources typically found in low-cost biomass feedstocks [19, 20]. Recently, great strides have been made to enhance the genetic tractability of *R. toruloides*, opening the door to a broad host of bioengineering applications [21, 22]. *R. toruloides* has been engineered to produce several bioproducts including 1,8-cineole, *ent*-kaurene, epi-isozizaene, prespatane, bisabolene, and the non-ribosomal peptide indigoidine [1, 19, 20, 23–26].

Most notably, *R. toruloides* is well suited for use in consolidated bioprocessing. The valorization of feedstocks into biocompounds typically involves segregating each step into individual unit operations including pretreatment, saccharification, microbial fermentation, and product separation. Consolidating these processes into as few operations as possible is critical for cost effectiveness [27–29]. We have previously demonstrated the robust capacity for *R. toruloides* to convert unfiltered lignocellulosic sorghum hydrolysates in a one-pot process into the biofuel precursor bisabolene [26], circumventing the high costs associated with solid-liquid separation. We have since shown that the use of ensiled biomass (a common storage method used by farmers around the world) in lieu of traditionally used field-dried biomass improves this one-pot process even further, reducing both the carbon footprint and minimum selling price of biofuels produced from *R. toruloides* [30]. During the ensiling process, the moisture that arises during storage enables substantial microbial organic acid fermentation [31]. This results in particularly high concentrations of acetic and lactic acids [30], the former of which is a strongly preferred carbon source for *R. toruloides* [32].

Furthermore, these acids have been used as components of a unique class of solvents called ionic liquids (ILs) or deep eutectic solvents (DESs), both of which have been used for pretreatment of lignocellulosic biomass to reduce the recalcitrance to enzymatic deconstruction of plant polysaccharides into fermentable sugars. Typically, pretreatment of lignocellulosic feedstocks involves first synthesizing the IL in an independent reaction, then adding the solvent to biomass. However, we have recently invented a novel one-pot deconstruction process that takes advantage of the organic acids released during ensiling to remove this unit operation entirely [33]. This “in-situ” IL synthesis process simplifies the procedure, potentially reducing operation and supply costs. While this is particularly useful for deconstructing ensiled biomass, dried biomass feedstocks (that do not contain appreciable levels of free organic acids) can be supplemented with organic acids to facilitate this reaction.

Here, we demonstrate the potential for *R. toruloides* to be used as a conversion host to valorize low-cost lignocellulosic feedstocks into valuable bioproducts such as TAL in a consolidated IL synthesis, pretreatment, saccharification, and fermentation process. We first engineer the yeast to produce 2.5 g/L TAL in bench-scale experiments, titers that are comparable to the highest titers achieved with substantial optimization efforts in *S. cerevisiae*. We subsequently integrate this strain into an advanced, separations-free one-pot bioreactor setup to convert sorghum hydrolysates into 3.9 g/L TAL. This work further establishes *R. toruloides* as a host for lignocellulosic biomass valorization, and identifies hurdles that will need to be overcome to fully realize the industrial potential of this advanced biorefinery strategy.

## Results and discussion

### Engineered *R. toruloides***produces substantial TAL**

**Fig. 1:**
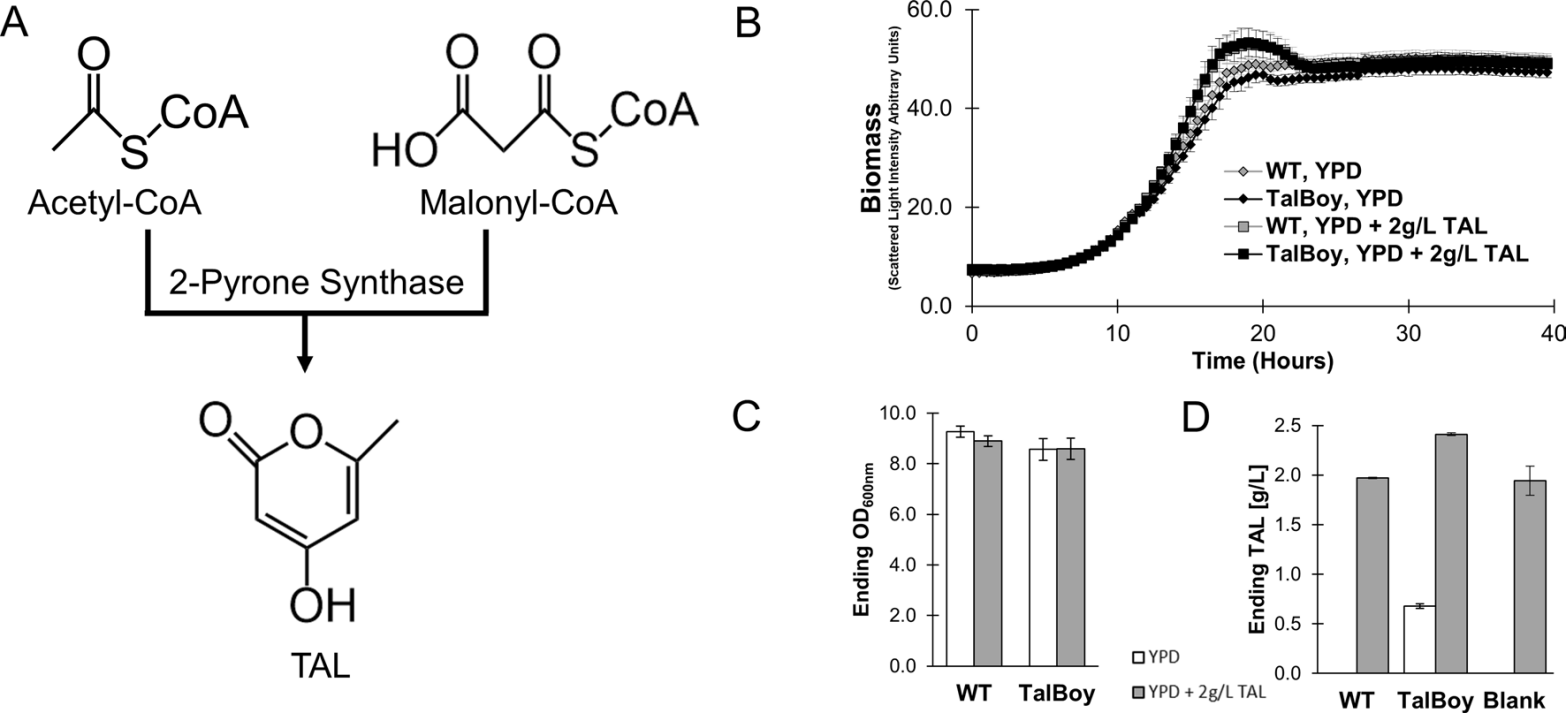
Establishing TAL production in *R. toruloides*. **A** Schematic of TAL production using 2-Pyrone Synthase (2-PS) from *Gerbera hybrida*. *2-PSG* was randomly integrated into the genome of *R. toruloides* IFO0880 (WT) to generate a TAL producing strain. **B** Growth of WT and TAL producing *R. toruloides* in YPD, or YPD supplemented with 2 g/L TAL, as tracked in a BioLector Pro microbioreactor. **C** Optical densities measured at the end of cultivation in the microbioreactor. **D** Titers of TAL recovered at the end of growth in **B** during growth in YPD (white bars), or YPD supplemented with 2 g/L TAL (grey bars). No TAL was observed from WT samples grown in YPD, nor was TAL observed in blank YPD. Error bars represent standard deviation of nine biological replicates (*R. toruloides* cultures) or three blank replicates (Blank)

We explored TAL production in *R. toruloides* by introducing the one-step pathway for converting malonyl-CoA and acetyl-CoA into TAL (Fig. 1A). We codon-optimized the 2-Pyrone Synthase (2-PS) gene from *Gerbera hybrida* (*2-PSG*) for expression in *R. toruloides*, and integrated it into the yeast’s genome. We selected an individual transformation isolate, and performed an initial screen to observe TAL production. Biological replicates of this strain were grown in standard YPD media, and were found to produce 1.65 ± 0.05 g/L of TAL. Notably, these initial titers are on-par with some of the highest reported titers achieved in *S. cerevisiae*, obtained after substantial targeted pathway interventions [8].

To ensure that TAL was neither degraded natively by *R. toruloides*, nor negatively influenced growth, we cultured WT and TAL producing *R. toruloides* in YPD, as well as YPD supplemented with 2 g/L TAL. Online growth was tracked using FlowerPlate microplates (with flower-shaped baffling to improve sample aeration) grown in a BioLector Pro for three days. We saw no significant differences in growth profiles between either strain in either culture condition (Fig. 1B). There were also no substantial differences in ending optical densities (Fig. 1C). We further quantified the TAL in each culture at the end of the experiment (Fig. 1D). In pure YPD, only TAL producing *R. toruloides* cultures contained any measurable TAL at the end of the experiment (0.68 ± 0.02 g/L). In YPD supplemented with TAL, we found no difference between the remaining amount of TAL in blank cultures (1.94 ± 0.15 g/L) not inoculated with cells, and cultures of WT *R. toruloides* (1.97 ± 0.01 g/L, *P* = 0.53). With this 2 g/L TAL supplementation, TAL producing *R. toruloides* cultures ended with 2.41 ± 0.01 g/L. This is less than the additive amounts of TAL supplementation and TAL produced in YPD (2 + 0.67 = 2.67), suggesting a slight feedback inhibition effect.

### Optimization of culture conditions for TAL production in a synthetically defined media

**Fig. 2:**
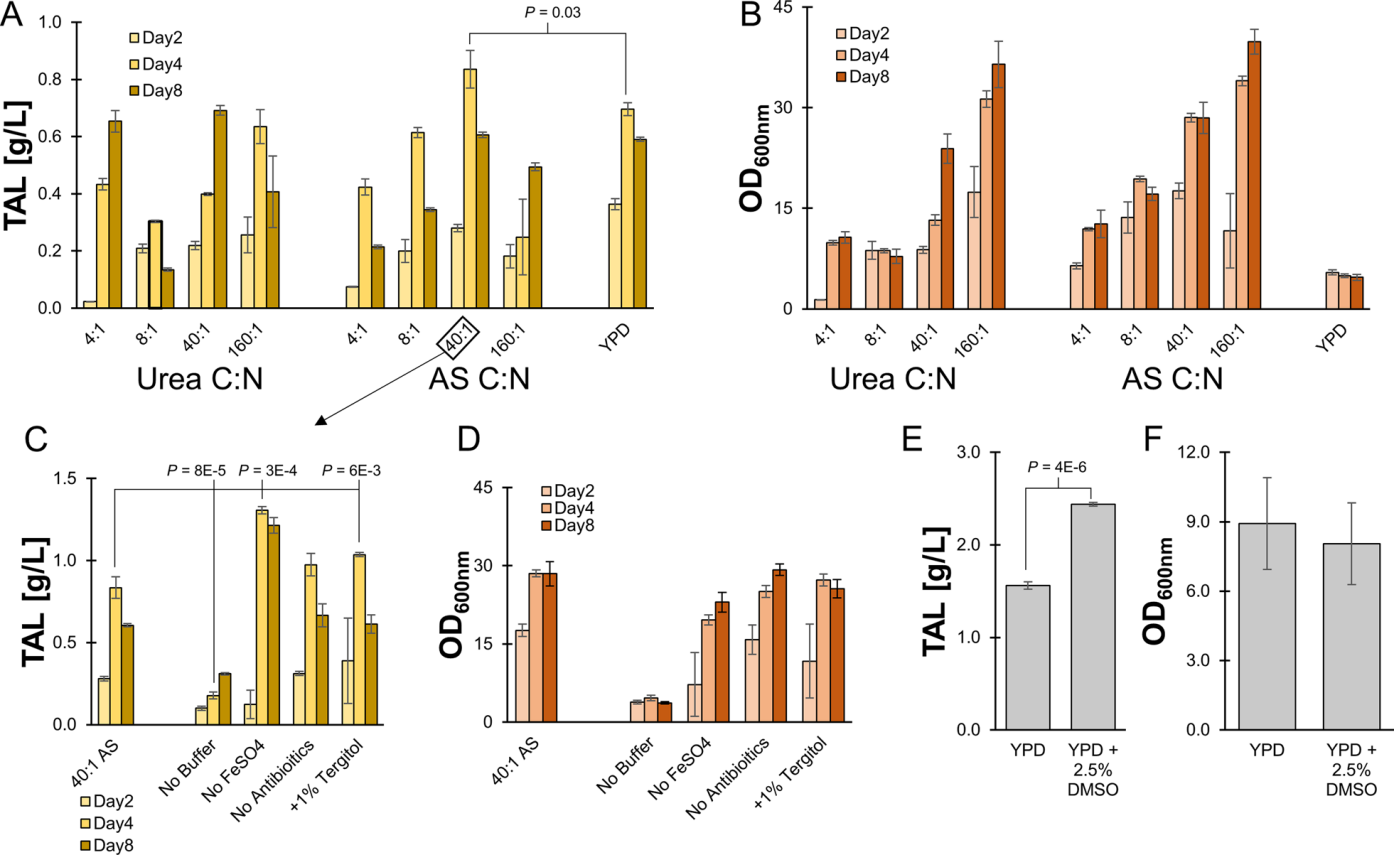
Optimization of media conditions to enhance TAL production in synthetic media containing 98 g/L glucose and 52 g/L xylose, as well as YPD (20 g/L glucose) as a control. A range of carbon to nitrogen (C:N) ratios were explored using urea or ammonium sulfate (AS) as the nitrogen source, by varying the amount of nitrogen source added. **A** Titers of TAL obtained from culture supernatant after two, four, and eight days of fermentation. **B** Growth of cultures in **A**, as measured by optical densities. **C** Comparisons of variations in experimental setup from the 40:1 C:N ratio using AS as the nitrogen source, in which the noted modification in the x-axis was made for each condition. **D** Growth of cultures in **C**, as measured by optical densities. **E** Comparison of titers in YPD media, and YPD supplemented with 2.5% DMSO, after five days of fermentation. **F** Optical densities of cultures in **E**. All error bars represent standard deviations of biological triplicates. *P*-values were calculated using a two-tailed type II Student’s *t-*test

One of the attractive aspects of *R. toruloides* is its ability to co-utilize multiple carbon sources, such as those found in hydrolyzed biomass. To explore the optimal conditions for production of TAL from *R. toruloides* from such feedstocks, we explored various culture conditions using complex synthetic defined media mixed with glucose and xylose (the two most prevalent carbon sources of hydrolyzed biomass) as the main carbon source. This allowed us to explore the influence of nitrogen addition (a necessary step in all *R. toruloides* fermentation of hydrolysate) and other culture conditions in a controlled manner, before moving on to exploring TAL production in hydrolysate. We first examined the influence of nitrogen sources and concentration, using either urea or ammonium sulfate in synthetic defined media. Varying the carbon to nitrogen ratio is known to substantially influence acetyl-CoA and malonyl-CoA availability, and thus the total TAL generated. Carbon to nitrogen ratio ranged from 4:1 to 160:1. Substantial loss in TAL titers were observed between days four and eight, likely due to TAL’s inherent long-term instability in solution as previously observed [10]. Optimal TAL titers of 0.84 ± 0.07 g/L occurred after four days of growth with ammonium sulfate C:N ratio of 40:1, with diminishing titers at both higher and lower C:N ratios (Fig. 2A). This was a slight but statistically significant increase in the equivalent titers obtained in YPD (0.70 ± 0.02 g/L, *P* = 0.03). In all other conditions on Day 4, titers were either statistically equivalent to YPD titers (160:1 C:N ratio of Urea) or statistically lower than YPD titers (P < 0.05). This is despite the substantially higher optical densities seen in these conditions (Fig. 2B). These results were reproducible in a second experiment (Additional file 1: Fig. S1).

To further optimize the synthetic defined media, we explored adjustments to the 40:1 C:N ammonium sulfate condition (Fig. 2C). We found that excluding the pH buffer drastically diminished TAL titers, likely because of a lack of growth (Fig. 2D) due to runaway lowering of pH. Addition of buffer to YPD also slightly improved titers, although these effects were minimal (Additional file 1: Fig. S2). Conversely, we found that the exclusion of FeSO_4_ increased TAL titers 1.6-fold to 1.31 ± 0.02 g/L (*P* = 3E-4). The addition of FeSO_4_ positively impacted production of ɑ-bisabolene in previously engineered *R. toruloides* [34]. That its inclusion negatively impacted titers points to the complex interaction between micronutrients and productivity that can change with each new pathway explored.

Finally, the addition of 1% Tergitol also slightly increased titers 1.3-fold to 1.04 ± 0.01 g/L (*P* = 6E-3). The addition of Tergitol to *R. toruloides* cultures has previously shown to increase fatty alcohol titers by promoting the export outside of the cell, thereby reducing metabolic burden and enabling further production [35]. A similar mechanism could have occurred here to enhance titers. In this vein of thought, we explored the addition of small amounts of DMSO to YPD to enhance titers to enhance TAL solubility and export. TAL is substantially more soluble in DMSO than in water, where the solubility limit is relatively low (approximately 8.6 g/L at 20 °C) [36, 37]. Furthermore, DMSO is known to enhance the membrane permeability of fungi [38]. Indeed, inclusion of 2.5% (by volume) DMSO significantly enhanced titers 1.6-fold to 2.44 ± 0.02 g/L (*P* = 4E-6) after five days of culturing (Fig. 2E). No impact on cell growth was observed (Fig. 2 F). We also explored 5% DMSO, although no growth was observed in this condition.

We briefly explored the effect of various antibiotic additions to YPD on growth and TAL production (Additional file 1: Fig. S3). While growth was somewhat hindered by addition of G418 in the first day of growth, this did not result in any differences in final TAL titers.

### *R. toruloides***supports valorization of the lignocellulosic feedstock sorghum into TAL**

While the production of gram per liter titers of TAL in a synthetic media supplemented with pure sugars is a promising sign for the industrial productivity of *R. toruloides*, such a media would likely not be commercially viable in an industrial setting due to the excessive costs of pure sugars. We therefore chose to next focus on demonstrating the use of this strain for TAL production from low-cost biomass feedstocks, by transitioning from the fermentation of laboratory-optimized media towards fermentation using deconstructed lignocellulosic biomass. We have shown that pretreatment of sorghum with the ionic liquid (IL) cholinium lysinate ([Ch][Lys]) in a consolidated “one-pot” process combined with enzymatic saccharification to effectively deconstruct plant matter into biocompatible, sugar-rich slurries [39, 40]. Importantly, this means that hydrolysates prepared from such pretreatment regimens can be directly employed for fermentation without costly upfront IL recycling operations [41, 42].

We sought to apply those learnings here, by exploring production of TAL from *R. toruloides* in hydrolysate derived from cholinium lysinate pretreatment of field-dried sorghum feedstocks. After hydrolysis, the resulting media was pH adjusted to 7.0 and filter sterilized to remove residual solids for subsequent fermentation with *R. toruloides.* This hydrolysate contained 76.6 g/L glucose and 31.9 g/L xylose. Supplementation of nitrogen is required for robust microbial growth in hydrolysates. Previously, we have demonstrated an optimal nitrogen source recipe for optimal production of PKS products from hydrolysates in the bacteria *Streptomyces albus* using yeast extract with supplementation, in a formulation designated “MM042” [43]. We applied this formulation here, while also testing yeast extract which is typically used as the nitrogen source. Both MM042 and yeast extract were provided as the nitrogen source, using a carbon to nitrogen ratio of 20:1 and 40:1.We also again explored the addition of 1.25% DMSO to enhance TAL titers, lowered from the previously used 2.5% DMSO to avoid the stress of high DMSO concentrations.

**Fig. 3:**
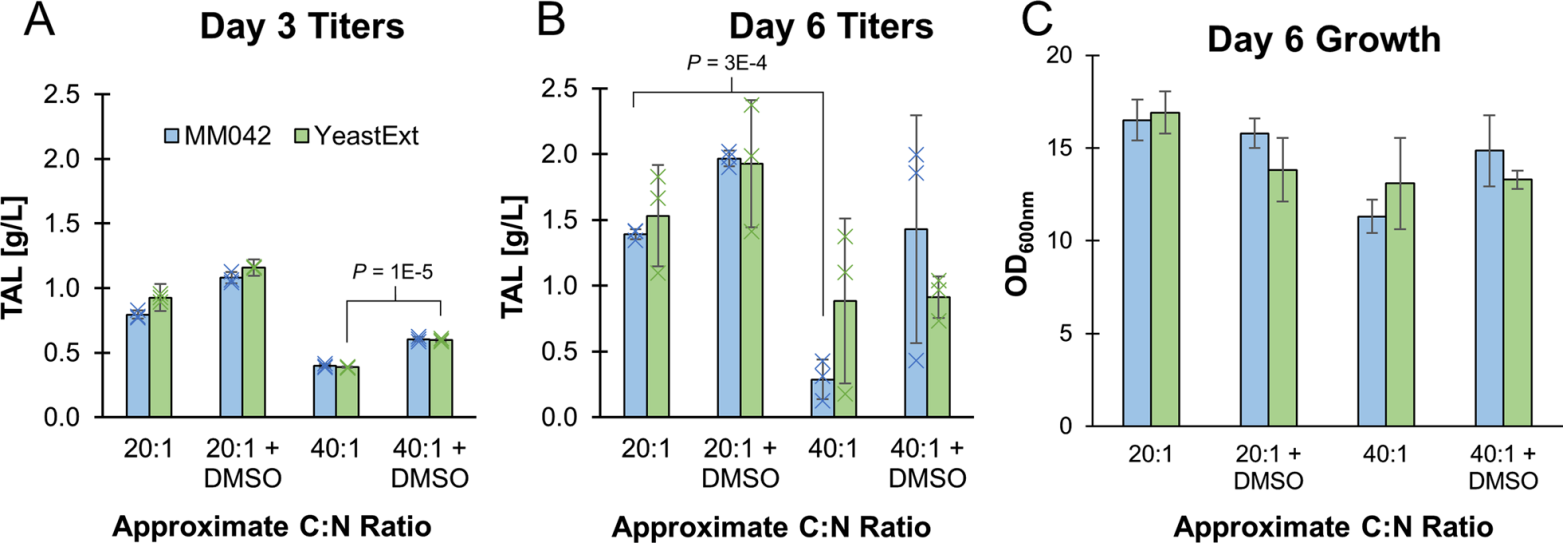
Production of TAL from *R. toruloides* in hydrolysates derived from dried sorghum biomass. **A** Titers obtained after three days of fermentation. The nitrogen source used (either MM042 or yeast extract) is listed, with the approximate molar ratio of carbon to nitrogen (assuming glucose and xylose as the primary carbon sources, and yeast extract as the nitrogen source). Cultures were grown in both the absence and presence of 1.25% v/v DMSO as indicated. **B** Titers obtained after six days of fermentation. **C** Optical densities after six days of growth. All error bars represent standard deviations of biological triplicates. *P*-values were calculated using a two-tailed type II Student’s *t-*test

By day three of fermentation, we observed significant TAL titers under all conditions explored (Fig. 3A). However, no differences in titers were observed between using either yeast extract or MM042 as the nitrogen source, indicating that the supplementations provided in MM042 had negligible impacts. For instance, the TAL titers obtained using a C:N ratio of approximately 40:1 with MM042 as the nitrogen source (0.40 ± 0.01 g/L) was statistically indistinguishable (*P* = 0.21) from titers obtained using plain yeast extract (0.39 ± 0.01 g/L). The lack of difference between MM042 and yeast extract as a nitrogen source was also true of the 20:1 C:N conditions, and whether or not DMSO was added to the media.

We did find that the C:N ratios substantially altered TAL titers, consistent with our earlier results. Lowering C:N ratios from approximately 40:1 to 20:1 resulted in significantly higher titers. Using MM042 as the nitrogen source, TAL titers were increased from 0.40 ± 0.01 to 0.79 ± 0.03 g/L, representing a 2.0-fold increase (*P* = 4E-5). These results held with yeast extract, where TAL titers increased from 0.39 ± 0.01 to 0.93 ± 0.03 g/L, representing a 2.4-fold increase (*P* = 9E-6). This occurred despite the slightly higher sugar content in the ~ 40:1 C:N ratio (61.2 g/L glucose and 22.3 g/L xylose) relative to the ~ 20:1 C:N ratio (53.5 g/L glucose and 21.9 g/L xylose). No other substantial sources of carbon equivalent to glucose or xylose were observed in the hydrolysate.

We also again found that the addition of DMSO enhanced TAL titers. Adding DMSO to the 20:1 C:N conditions increased titers 1.36- and 1.25-fold using MMO42 or yeast extract respectively (*P* = 8E–4 and *P* = 2E-4 respectively). These results held at the 40:1 C:N conditions, where DMSO addition increased titers 1.51- and 1.54-fold using MMO42 or yeast extract respectively (*P* = 1E–4 and *P* = 1E-5 respectively). These trends held by day six of the experiment (Fig. 3B). In the best case scenario using MM042 as the nitrogen source, a C:N ratio of approximately 20:1, and adding DMSO, we obtained 1.97 ± 0.06 g/L TAL. However, we did observe signs of TAL degradation at later time points, indicating that TAL stability may decrease in longer fermentations.

No substantial differences in growth were observed at the experiment end (Fig. 3C). There was a slight increase in the observed cell density in MMO42 media using a 20:1 C:N ratio (OD_600_ = 16.5 ± 1.1) versus a 40:1 C:N ratio (OD_600_ = 11.3 ± 0.9, *P* = 2E-3), but these results are largely minimal.

### **Ensiling sorghum enhances***R. toruloides***TAL production from lignocellulosic feedstock**

Farmers typically have two options after harvesting grassy biomass: letting it dry in the field followed by bailing, or ensiling, which entails storing wet biomass under aerobic conditions until such a time as biomass can be used. This often leads to anaerobic bacterial lactic acid fermentation, producing substantial quantities of low molecular weight organic acids. The production of acetic acid is of particular interest, as *R. toruloides* is known to utilize up to 20 g/L acetic acid as the sole carbon source [32], and exploiting acetic acid’s redox-related impact on metabolism was fundamental to enhancing TAL titers in *Y. lipolytica* [12]. We have shown that *R. toruloides* fermentation of ensiled biomass is economically and environmentally preferable [30]. We therefore next explored the production of TAL from *R. toruloides* fermentation on hydrolysates produced from sorghum that was either field-dried (“Dry”) or field-ensiled (“Ensiled”), using the optimal C:N ratio discovered in our previous experiments for Dry hydrolysate (~ 20:1 C:N, based upon glucose and xylose concentrations and using yeast extract without supplementation).

Both field-dried and ensiled sorghum feedstocks were deconstructed in parallel using one-pot cholinium lysinate pretreatment as previously outlined. A subset of these raw hydrolysates were filtered, and used for fermentation with *R. toruloides* into TAL. The remaining portion of this batch was kept unfiltered, and used for future experiments. Over twice as much glucose was present in the hydrolysates produced from ensiled biomass (27.0 ± 1.3 g/L dry, 59.6 ± 0.9 g/L ensiled), as well as over three fold xylose (5.8 ± 0.6 g/L dry, 21.1 ± 0.7 g/L ensiled). Additionally, substantially amounts of acetic acid (21.1 ± 0.3 g/L) and lactic acid (17.8 ± 0.2 g/L) were present in hydrolysate from ensiled biomass, while only trace amounts (0.8 ± 0.1 and 2.09 ± 0.01 g/L of acetic and lactic acid respectively) were present in the hydrolysate from dry biomass. The lower sugar content overall in the dry hydrolysate compared to our previous experiment is likely due to lower solid biomass loading, as well as batch-to-batch variation in hydrolysate preparation.

We next explored the ability of the engineered strain to convert these deconstructed feedstocks into TAL. As we had not explored the effect of the additional organic acids on C:N ratio optimization, we again prepared our nitrogen supplementation using yeast extract, and glucose and xylose concentrations to approximate a starting C:N ratio of 20:1 for each hydrolysate independently.

**Fig. 4:**
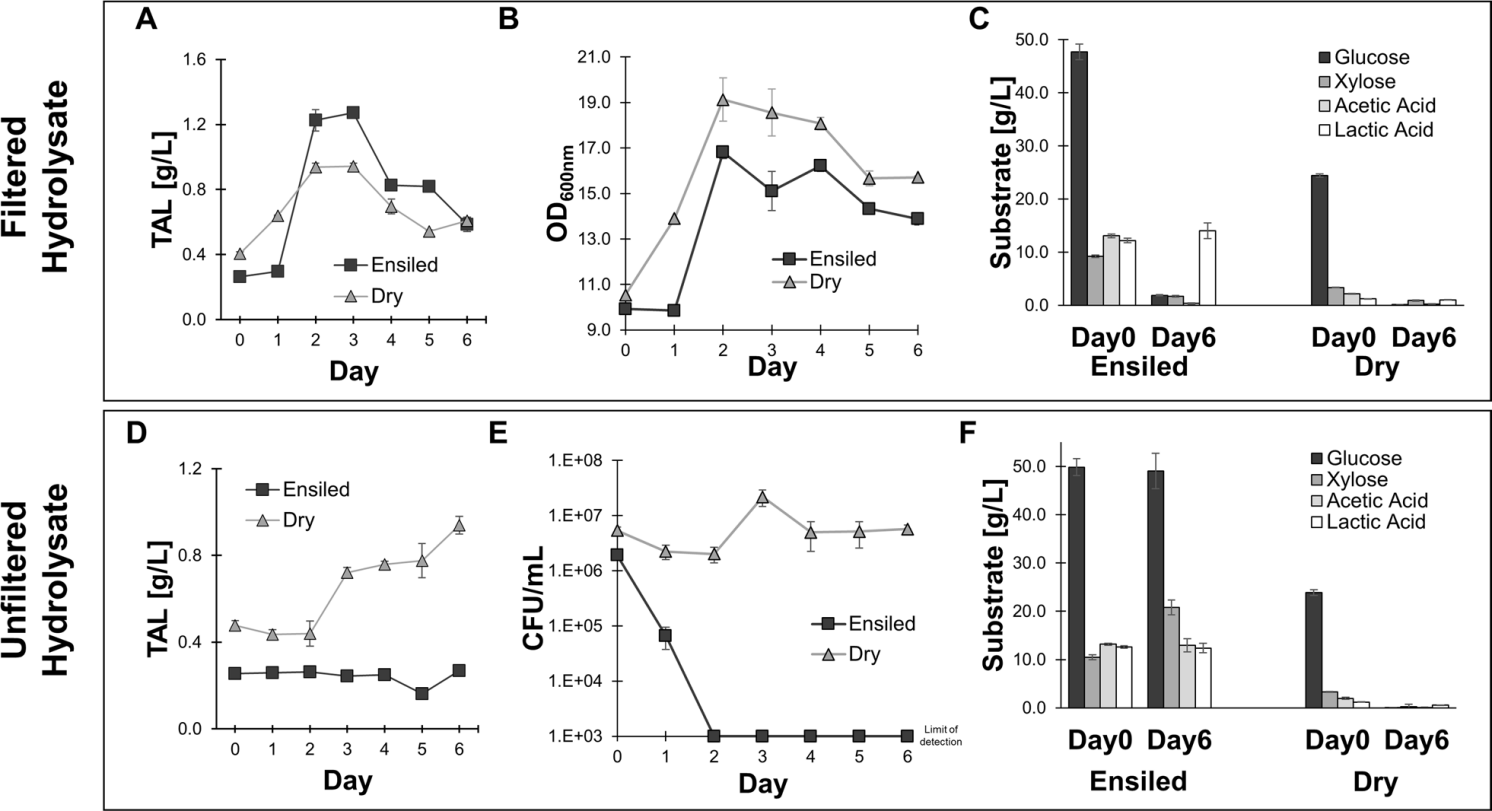
Comparison of dried/ensiled hydrolysates for TAL production, both with and without filtering hydrolysates prior to fermentation. **A** Titers obtained during fermentation on filtered hydrolysates over time. **B** Growth in filtered hydrolysates, as measured by optical densities. **C** Primary carbon sources during fermentation on filtered hydrolysates, at the experiment start and end. **D** Titers obtained during fermentation on unfiltered, pasteurized hydrolysates over time. **E** Growth in unfiltered hydrolysates, as measured by viable colony forming units (CFUs) of *R. toruloides* on YPD plates. The limit of detectable colony units is indicated (1000 units/mL). **F** Primary carbon sources during fermentation on unfiltered hydrolysates, at the experiment start and end. All error bars represent standard deviations of biological triplicates

In the first day of the experiment, TAL production was significantly greater (*P* = 8E-6) in the dry hydrolysate (0.639 ± 0.003 g/L) than in the ensiled hydrolysate (0.30 ± 0.02 g/L) (Fig. 4A). This is likely due to no measurable growth in this same timeframe in the ensiled hydrolysate, indicating a slight lag phase prior to growth (Fig. 4B). However, by day two, both growth and TAL production ramped up in the ensiled hydrolysate. By day 3, TAL production peaked, with TAL titers significantly greater (*P* = 7E-5) in the ensiled hydrolysate (1.27 ± 0.03 g/L) than the dry hydrolysate (0.94 ± 0.02 g/L). The vast majority of sugars were consumed by *R. toruloides* in both hydrolysates throughout the experiment (Fig. 4C). Likewise, the substantial acetic acid present in the ensiled hydrolysate was also nearly fully consumed. Interestingly, no lactic acid was consumed, despite our previous findings that *R. toruloides* is capable of consuming lactic acid in hydrolysates prepared from cholinium lysinate [26].

Collectively, these results suggest that ensiled biomass can serve as a superior feedstock for maximizing the amount of bioproduct produced. In a separate experiment using dry and ensiled hydrolysates produced in a separate batch, we again saw significantly more (*P* = 2E-5) TAL production in ensiled hydrolysate (2.50 ± 0.06 g/L) than in dry hydrolysate (1.58 ± 0.03 g/L) (Additional file 1: Fig. S4).

We also explored the addition of 1.25% DMSO to enhance TAL production from these Ensiled hydrolysates, as we observed earlier in Dry hydrolysates (Additional file 1: Fig. S5). While we did observe an increase in TAL titers from 2.7 to 2.9 g/L, this increase was rather small and not statistically significant (*P* = 0.11). Furthermore, it was associated with a substantial delay in growth and TAL production. To avoid the possibility of further negative impacts of DMSO addition, we excluded DMSO in further experiments.

### *R. toruloides* supports separations-free fermentation of dry but not ensiled biomass

In the conventional biorefinery setup, the solid-liquid slurry produced from biomass pretreatment is typically filtered before downstream fermentation into the desired products. This involves a clarification process operation to remove solid particulates from the slurry (typically membrane filtration), a process that is laborious and can significantly increase costs [44, 45]. Removing this step is therefore an attractive alternative to reducing costs. We have previously demonstrated the robust capacity for *R. toruloides* to directly ferment unfiltered hydrolysates without filtration into the biofuel bisabolene [26], resulting in a more advanced “one-pot” process combining not only pretreatment and saccharification, but fermentation as well.

We therefore sought to next determine if *R. toruloides* could efficiently ferment unfiltered hydrolysates from dry and ensiled sorghum feedstocks into TAL. For this, we utilized the remaining hydrolysate produced from the same batch of dried and ensiled sorghum used to produce data for Fig. 4A–C, but was left unfiltered earlier. To sterilize this hydrolysate, we pasteurized samples at 80 ℃ for one hour and cooled to room temperature prior to fermentation.

In contrast to our previous results using filtered hydrolysates, we found that *R. toruloides* failed to produce any TAL in unfiltered ensiled hydrolysate (Fig. 4D). TAL titers did not change appreciably throughout the course of the experiment. As the significant solid concentrations precluded our measurement of cell growth using optical densities, we instead directly quantified total viable *R. toruloides* cells by measuring colony forming units (CFUs). We found that *R. toruloides* cells rapidly died in this unfiltered ensiled hydrolysate, dropping from 1.9 ± 0.4 E6 CFU/mL at the start of the experiment to below the detectable limit by day two (Fig. 4E). As a result, there was virtually no change in the concentration of sugars or organic acids throughout the course of the experiment in unfiltered ensiled hydrolysate (Fig. 4F).

Conversely, *R. toruloides* was perfectly capable of producing TAL in unfiltered dry hydrolysate. Although both growth and production were delayed in the first two days of the experiment, by the third day we observed substantial TAL production and growth. TAL titers peaked on day four, at 0.94 ± 0.04 g/L. Again, virtually all carbon sources had been consumed by the end of the experiment in unfiltered dry hydrolysate.

This experiment using both filtered and unfiltered hydrolysates was also performed in parallel with the same conditions, but only allowing for one day of adaptation to hydrolysate in the seed train (as opposed to the two days used here). The trends observed here were recapitulated with no notable differences, indicating that a shorter seed train could be used (Additional file 1: Fig. S6). The fact that there was a growth delay in the unfiltered dry sorghum hydrolysate indicates that the particles have a mild inhibitory effect. The filtered ensiled hydrolysate also caused a growth delay, so perhaps in combination with a particle-driven growth defect, the environment was too inhibitory for the organism to grow in the unfiltered ensiled hydrolysate. Further investigation and process optimization is needed to understand why the particles mildly inhibit growth and develop methods to alleviate this inhibition.

### In-situ synthesis of IL in one-pot pretreatment

Typically, pretreatment of feedstocks with cholinium lysinate involves first synthesizing the IL in an independent reaction of choline hydroxide with L-lysine, before addition to biomass slurries to initiate pretreatment. However, we have shown that the organic acids produced in ensiled biomass (or supplemented to dry biomass) allows us to remove this unit operation entirely by synthesizing IL/DESs in-situ (in the biomass pretreatment reaction) [33]. This “in-situ” IL synthesis process simplifies the procedure, potentially reducing operation and supply costs.

To demonstrate the feasibility of this advanced one-pot deconstruction regimen, we next employed in-situ IL synthesis to pretreat both dry and ensiled sorghum. Due to the poor growth in unfiltered ensiled hydrolysates produced from traditional IL synthesis, we focused on the use of filtered hydrolysates. As organic acids are essential for in-situ IL formation, and are largely absent in dried biomass, acetic acid and lactic acid were added to the pretreatment reactions of dry hydrolysate at concentrations similar to those observed in ensiled biomass slurries (13.3 ± 0.3 g/L acetic acid and 12.9 ± 0.2 g/L lactic acid, respectively). We again found that ensiled biomass produced more sugars than dry biomass after pretreatment. The hydrolysate from ensiled biomass prepared using in-situ pretreatment produced 35.3 g/L glucose and 17.6 g/L xylose, while the same prepared from dry biomass produced 27.1 g/L glucose and 15.1 g/L xylose. This corresponds to higher starting sugars in fermentation with *R. toruloides*; cultures using ensiled feedstock began with 35.3 ± 3.7 and 17.6 ± 1.7 g/L glucose and xylose respectively, while those using dry feedstock began with 27.1 ± 1.2 and 15.1 ± 0.8 g/L glucose and xylose respectively.

**Fig. 5:**
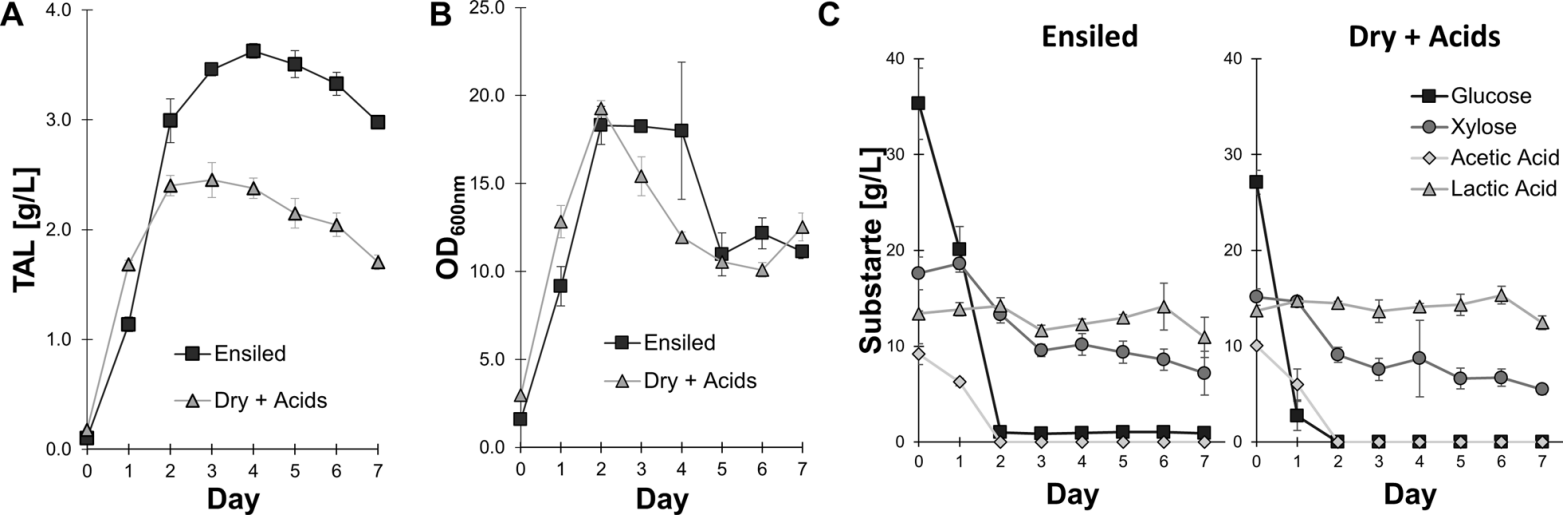
Use of one-pot pretreatment and saccharification, in which the IL is synthesized “in-situ” during biomass deconstruction. Filtered-sterilized hydrolysates were prepared from either ensiled sorghum, or dry sorghum with equivalent acetic and lactic acids added to facilitate in-situ IL synthesis. **A** TAL titers obtained during *R. toruloides* fermentation on each hydrolysate. **B** Growth in each hydrolysate during fermentation. **C** Sugars and acids during fermentation. Acetic and lactic acid in dry hydrolysate was artificially supplemented to match the levels observed in ensiled. All error bars represent standard deviations of biological triplicates

Hydrolysate from ensiled biomass again proved better for enhancing TAL titers during fermentation with *R. toruloides*. A maximum titer of 3.63 ± 0.07 g/L TAL was obtained on the fourth day of fermentation on ensiled hydrolysates produced from in-situ pretreatment, significantly higher than the maximum titer of 2.45 ± 0.16 g/L TAL obtained on the third day of fermentation on equivalent hydrolysate from dried sorghum (Fig. 5A). These titers are notably higher than those obtained from any hydrolysate made from traditional one-pot processes. Negligible growth differences were observed between fermentation on either hydrolysate, with ODs peaking on the second day of fermentation (Fig. 5B). This corresponds with a near total consumption of glucose and acetic acid by day two (Fig. 5C). While xylose concentrations reduced substantially during fermentation in both ensiled (17.6 ± 1.7 g/L starting, and 7.2 ± 2.3 g/L ending) and dry (15.1 ± 0.8 g/L starting, and 5.5 ± 0.5 g/L ending) hydrolysates, it was not fully consumed in either fermentation. Likewise, lactic acid concentration changed very little in both ensiled (13.4 ± 0.3 g/L starting, and 10.9 ± 2.1 g/L ending) and dry (13.7 ± 0.6 g/L starting, and 12.5 ± 0.7 g/L ending) hydrolysates.

Collectively, these results demonstrate that the one-pot fermentation and saccharification process can be further consolidated by including the synthesis of cholinium-based ILs in-situ within the reaction.

### One-pot IL synthesis, pretreatment, saccharification, and bioreactor fermentation

We next sought to combine the lessons learned from previous experiments in a bioreactor fermentation setup, to explore the industrial potential of this process. While we have consistently demonstrated the use of ensiled feedstocks improves performance when hydrolysates are filtered, the use of unfiltered ensiled sorghum clearly inhibits growth of *R. toruloides* more than unfiltered dry sorghum through an unknown mechanism. We therefore utilized unfiltered hydrolysate derived from dry sorghum produced through in-situ IL pretreatment in this scale-up experiment. We performed biomass deconstruction and microbial fermentation in separate vessels, but these unit operations could be combined in a single vessel in future iterations of this process.

Hydrolysate prepared through one-pot in-situ pretreatment and saccharification of dry sorghum (supplemented with 6.3% (v/v) acetic acid and 5.8% (v/v) lactic acid, equivalent to concentrations found in ensiled hydrolysate) contained 37.6 ± 1.4, 19.7 ± 1.6, 15.4 ± 0.2, and 15.2 ± 0.6 g/ L of glucose, xylose, acetic acid, and lactic acid respectively. Glucose and xylose were again used to estimate a starting C:N ratio of 20:1, and fermentation performed in a 2 L Sartorius bioreactor setup. In our first attempt of this experiment, substantial foaming occurred within 48 h despite copious amounts of antifoaming agent being added. To combat this foaming, an overlay of 20% (v/v) dodecane was applied, as we have consistently found this to substantially decrease foaming in the past [20, 26, 35, 40]. Additionally, we found the partition coefficient of TAL in water: dodecane mixtures to be 1.11, indicating this overlay would have minimal impact on TAL solubility relative to other overlays tested (Additional file 1: Fig S7).

**Fig. 6:**
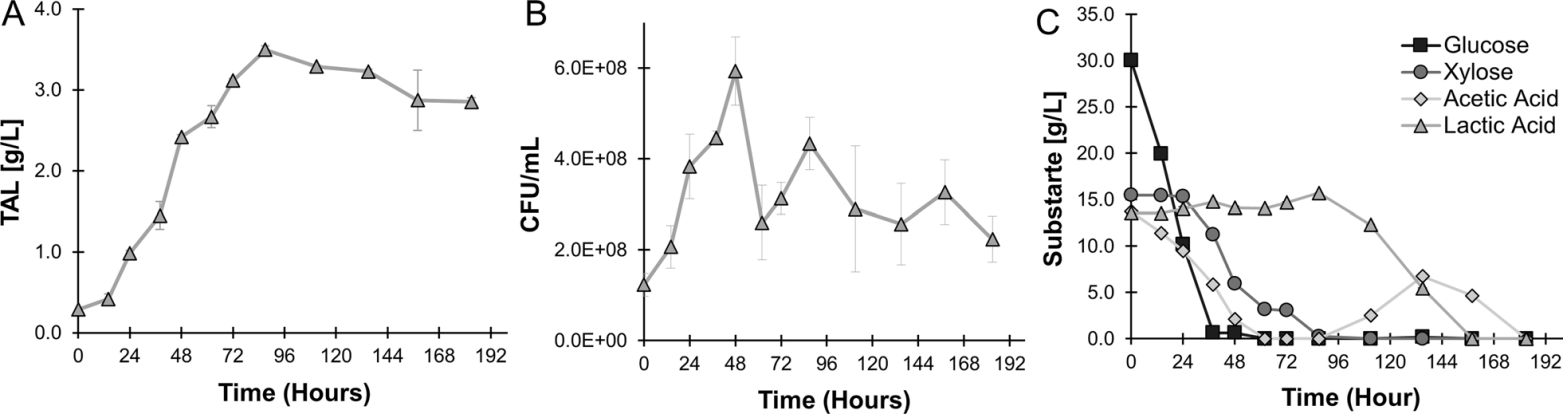
Production of TAL from *R. toruloides* in 2 L fermentations using unfiltered hydrolysate derived from dried sorghum, supplemented with acetic and lactic acids to enable in-situ IL synthesis during biomass pretreatment. **A** TAL titers during fermentation. **B** Growth during fermentation, as quantified by viable CFUs. **C** Sugars and acids during fermentation. All error bars represent standard deviations of technical triplicates

Using these conditions, bioreactor cultivation led to enhanced TAL production. Titers peaked after 87 h of fermentation, at 3.50 ± 0.05 g/L TAL in the aqueous layer (Fig. 6A). Taking into account the partition coefficient, 3.88 ± 0.05 g/L TAL was present in the dodecane overlay. Growth peaked after 48 h of fermentation, with total viable *R. toruloides* cells increasing 4.8-fold from the start of the experiment (Fig. 6B). Cell viability dipped briefly afterwards, which corresponded with the emergence of contaminating bacteria colonies. We have previously observed such contamination when working with unfiltered hydrolysates [26]. 16 S rRNA sequencing revealed this contamination to most directly match (99.4% homology) *Lysinibacillus macroides*, an endospore-forming bacteria known to consume lactic acid [46]. Furthermore, *bacilli* are known to facilitate conversion of lactic acid to acetic acid [47], which could explain the consumption of lactic acid and production of acetic acid late in fermentation (Fig. 6C). Our earlier data reveals an apparent lack of lactic acid consumption by *R. toruloides* under these experimental conditions, which is perplexing as this organism is known to consume lactic acid. However, it is possible that this contamination was actually beneficial for promoting the conversion of lactic acid into acetic acid, a carbon source that *R. toruloides* appears to prefer. We have recently shown that *R. toruloides* consumes acetic acid while avoiding succinic acid consumption in a proof-of-concept sequential bioreactor setup, supporting the notion of preferential organic acid consumption by *R. toruloides* from mixed-carbon feedstocks [48]. Despite this contamination, *R. toruloides* growth and production of TAL remained robust. This represents the highest titer of any fermentation product produced from *R. toruloides* using a separations-free process, and establishes the organism as an attractive host for equivalent industrial biorefinery system.

## Discussion

Across the world, researchers have put forth great efforts towards the engineering of microbes to produce higher and higher titers of valuable chemicals. Likewise, methods to enhance the yields and reduce the costs of deconstruction processes to pretreat biomass feedstocks into a form suitable for fermentation continue to be developed. To bring forth a robust bioeconomy in which value-added products are generated from renewable sources, these two efforts must be merged. This study lays forth how biomass deconstruction and subsequent microbial valorization can be consolidated into as few distinct processes as possible, while still producing industrially relevant titers of desired bioproducts.

The first part of this manuscript lays out our efforts to engineer *R. toruloides* to produce the commercially attractive polyketide, TAL. Much of the previous work towards bioengineering the production of TAL has focused on two model organisms: *E. coli* and *S. cerevisiae*. The introduction of the gene 2-pyrone synthase from *Gerbera hybrida* (the common daisy) into both of these organisms showed relatively high titers of 0.47 and 1.8 g/L for *E. coli* and *S. cerevisiae* respectively [9]. These titers in *E. coli* were improved through mutagenesis of the 2-pyrone synthase to 2.1 g/L, although it was noted that significant growth impairment occurred above approximately 1 g/L [49]. While efforts have continued to try and improve TAL titers in *E. coli*, these efforts tend to result in titers below a gram per liter, and have generally failed to keep pace with the titers observed in yeast [50]. Titers of TAL production from *S. cerevisiae* have reached 23.9 g/L, well above the compound’s solubility limit [36]. Although *S. cerevisiae* continues to be an attractive host for TAL production [51], other yeasts have become more promising hosts for TAL production in recent years. In particular, researchers have found the ascomycetous yeast *Y. lipolytica* a robust organism for the production of multiple grams per liter titers of TAL [5, 11]. Indeed, the highest TAL titers reported to date (35.9 g/L) come from this oleaginous yeast [12]. While these titers are quite high, they are the result of using refined substrates as the carbon sources for *Y. lipolytica* to assemble TAL from. Such titers would likely drop substantially if a more economically practical feedstock was supplied to the organism. Towards that end, *Y. lipolytica* is notoriously reluctant to catabolize xylose, the second most abundant carbon source in deconstructed biomass, particularly in feedstocks with mixed carbon sources [52]. The yeast also has trouble growing at higher concentration of furfural and formic acid, both of which are prevalent in hydrolysate derived from lignocellulosic biomass [53].

As the use of refined substrates are likely to be cost prohibitive in an industrial context, focus must also be given towards reducing the price of feedstock used by these organisms to serve as the building blocks of their final products. Notably, *R. toruloides* is able to utilize both glucose and xylose simultaneously [20], thereby presenting a distinct advantage over organisms such as *Y. lipolytica* to serve as a microbial host for valorizing feedstocks of mixed carbon sources. Furthermore, *R. toruloides* growth is also relatively robust in the presence of common inhibitors found in hydrolysate [54], while still maintaining many of the attractive qualities of *Y. lipolytica* such as its naturally high pools of acetyl and malonyl-CoA [20]. Here we show that by introducing 2-pyrone synthase into *R. toruloides*, we obtain titers of TAL (1.65 ± 0.05 g/L) similar to those first obtained in *Y. lipolytica* (approximately 1.5 g/L) [12]. Optimization of metabolic flux further enhanced these titers to 4.9 g/L, as would likely be expected if metabolic optimization were performed on our strain of TAL producing *R. toruloides*. Indeed, during preparation of this manuscript, another lab reported production of 23 g/L TAL in *R. toruloides* with substantial metabolic engineering efforts from the juice of engineered sugarcane feedstock, further underscoring the ability for *R. toruloides* to serve as an attractive host for TAL [55]. Furthermore, they noted no issues with TAL solubility or observed product precipitation at these high concentrations that are 2.6-fold above the solubility limit, a problem that has frequently plagued researchers when the production of TAL from their engineered yeast strains approaches the solubility limit [12, 36, 37, 56]. This suggests that TAL recovery from *R. toruloides* cultures for quantification was not a significant problem for these researchers.

The second portion of this manuscript focuses on methods to reduce the costs associated with converting biomass feedstock into a form suitable for fermentation by *R. toruloides* into the product we have engineered it to produce. Notably, the pretreatment of lignocellulosic feedstocks is estimated to be the most expensive step in the microbial production of commodities such as ethanol, accounting for roughly 20% of total costs [57]. A significant portion of these costs are associated with the cleanup of toxic inhibitory compounds from hydrolysates produced from biomass pretreatment [58]. By choosing an organism known for its ability to tolerate compounds typically found in hydrolysate at levels that are inhibitory to other organisms, we are able to minimize the costs associated with removing these compounds. Furthermore, *R. toruloides* is known to tolerate many ionic liquids used in pretreating biomass [59]. This allows us to also remove the steps involved with cleanup of ionic liquids prior to fermentation, thereby combining the steps in a single unit operation in a one-pot process. We have demonstrated this previously with *R. toruloides* production of the potential jet fuel, bisabolene [26]. However, this study consolidates the process even further by demonstrating that the ILs used to pretreat biomass feedstocks can be synthesized in-situ, in the same tank in which subsequent biomass deconstruction and microbial fermentation occurs, without the need of any separations step. To our knowledge, this is the first demonstration of in-situ IL synthesis, thereby making the process we outline here the most consolidated process to date.

While we were able to successfully generate 3.9 g/L TAL in the dodecane overlay with this process from dried biomass, we did note the presence of contaminating bacteria at the end of fermentation. This organism, likely *Lysinibacillus macroides*, is likely able to survive the relatively mild pasteurization conditions we employed (heating at 80 °C for one hour). Overcoming such contamination, such as alternative pasteurization techniques [60], will be required to establish a reliably reproducible separations-free fermentation strategy. From a different perspective, the fact that this process and organism is still able to produce sufficient amounts of TAL in the presence on an organism speaks to its robustness. *R. toruloides* can consume lactic acid, so overall, no carbon is lost when the contaminant is a lactic acid bacteria. Contamination is a common problem in industry, and this organism’s ability to eat organic acids makes contamination less of an issue relative to other processes that cannot convert these carbon sources. Additionally, while we demonstrated success with the use of dried biomass input, we were unable to apply the one-pot process to ensiled biomass (the storage strategy preferred by farmers) without including a step to separate the large particulates from the fermentable slurry. If there were some unidentified small molecule inhibitors unique to ensiled and not dried biomass (such as an antifungal produced by microbes during ensilage), one would expect that filtering the ensiled hydrolysate would not reduce its toxicity. However, as we see substantial growth and higher titers with ensiled biomass than dried biomass inputs when both are filtered, this points to some other explanation for the inability of *R. toruloides* to grow in unfiltered ensiled biomass. Identifying this reason will be critical if ensiled biomass is to be incorporated into the advanced one-pot deconstruction and valorization strategy outlined in this study. Finally, we note the slight decrease in TAL titers observed in Figs. 4, 5 and 6 after the conclusion of fermentation is likely not the result of *R. toruloides* consumption of TAL, but rather is possibly the result of the inherent instability of TAL in solution, as has been previously reported to occur at elevated pH levels [10].

## Conclusion

Here we engineer *R. toruloides* to produce the valuable polyketide TAL, and integrate this strain into an advanced one-pot process combining feedstock deconstruction and microbial valorization of lignocellulosic sorghum biomass into TAL. We obtain 3.9 g/L TAL via fermentation of this engineered *R. toruloides* strain on separations-free hydrolysate, representing the highest titers achieved from biomass for TAL. The advanced one-pot process used in this study takes advantage of the organic acids released during biomass ensilage to enable in-situ IL synthesis, further reducing the amount of unit operations necessary. The use of such low-cost feedstocks will be integral to establishing industrially relevant biorefinery strategies for generating polyketide-based products in both an economically and environmentally sustainable manner.

## Materials and methods

### Standard culture conditions

Cultures of *Escherichia coli* were inoculated from glycerol stocks into 5 mL lysogeny broth (LB) (Becton, Dickinson and Company, Franklin Lakes, NJ, USA) cultures in 50 mL glass conical culture tubes, and grown at 37 °C with 200 rotations per minute (rpm) shaking overnight. Cultures of *R. toruloides* strain IFO0880 and other strains of *R. toruloides* developed in this manuscript were inoculated from glycerol stocks into 5 mL Yeast Extract-Peptone-Dextrose (YPD) (Becton, Dickinson and Company) cultures in 50 mL glass conical culture tubes, and grown at 30 °C with 200 rpm shaking for 2 to 3 days.

### Plasmid and strain construction

The coding sequence of the *Gerbera hybrida* gene encoding 2-pyrone synthase (*g2ps1*, UniProt ID P48391*)* was codon-optimized by GenScript (Piscataway, NJ, USA) for optimal expression in *R. toruloides*. This sequence was placed under control of the TEF1 promoter of translational elongation factor 1, one of the strongest promoters known for *R. toruloides* which we have shown previously to drive high production of desired products [24]. The high efficiency 35 S gene terminator was used to terminate transcription. A G418R antibiotic resistance marker under the TUB2 promoter and terminators was used for antibiotic selection. These genes were flanked by 1 kb homology arms for homologous recombination of the construct at the *car2* locus of *R. toruloides*. This construct was synthesized by GenScript, to create plasmid pPBO106. This construct contains KanR for selection in *E. coli*. To recover plasmid, glycerol stocks of *E. coli* harboring pPBO106 were inoculated into 5 mL LB + Kanamycin (Sigma-Aldrich, St. Louis, MO, USA). Plasmid was recovered via a QIAprepSpin Miniprep Kit (Qiagen, Hilden, North Rhine-Westphalia, Germany).

Wild-type (WT) *R. toruloides* IFO0880 was obtained from glycerol stocks of the strain described in our previous study [20]. The plasmid pPBO106 was digested with PvuII-HF (New England Biolabs, Ipswich, MA, USA) and transformed into *R. toruloides* using lithium acetate heat shock as previously described [21]. G418 antibiotic selection (brand name Gibco™ Geneticin™) (Thermo Fisher Scientific, Waltham, MA, USA) was used to recover correct transformations, including both red and white colonies indicating random integration and targeted integration at the *car2* locus respectively. We decided to proceed forward with the randomly-integrated variant, as both targeted and randomly integrated variants proved to produce comparable levels of TAL and afforded the possibility of further downstream engineering using *car2* red/white screening. This strain, and the plasmid to construct it, are available upon request through the JBEI Private Registry. The final strain of *R. toruloides* that produces TAL can be requested using Part ID JBx_223021, while *E. coli* harboring the plasmid used to construct this strain (as well as sequencing data of this plasmid) can be requested using Part IDIT JBx_134470.

### Microplate cultivation conditions

For testing TAL toxicity and consumption by *R. toruloides*, engineered and WT *R. toruloides* were first inoculated from a glycerol stock onto a YPD 2% agar petri plate and grown for 3 days at 30 °C. Three individual colonies of engineered or WT *R. toruloides* were inoculated into 5 mL YPD in 50 mL glass culture tubes, and incubated for 2 days at 30 °C. For each of the 3 cultures, 10 µL of samples were then diluted into 3 replicates of 490 µL YPD or 490 µL YPD supplemented with 2 g/L TAL (Sigma-Aldrich) for a total of 9 replicates for each condition. Cultures were grown in a 48-well FlowerPlate (m2p-labs, Baesweiler, North Rhine-Westphalia, Germany) with a transparent bottom for online growth measurements, and a gas-permeable sealing film cover manufactured for reduced evaporation (m2p-labs). Cultures were grown at 30 °C with 975 rpm and 87.5% humidification in a BioLector Pro microbioreactor (m2p-labs). Growth measurements were tracked every 30 min, with measurements performed using the biomass filter set to a gain of two. This measures scattered light intensity, analogous to optical density measurements [61]. After 3 days of growth, ending optical densities were also measured by diluting samples 1:100 in water and using the 600 nm wavelength (OD_600_) of a SpectraMax Plus 384 Microplate Reader (Molecular Devices, San Jose, CA, USA). Samples were subsequently centrifuged at 4000 rpm for 5 min, the supernatant filtered through a 0.45 μm filter (Agilent Technologies, Santa Clara, CA, USA), and TAL concentrations quantified as described later.

For exploring optimal carbon to nitrogen ratios, a similar experimental setup was performed. In this case, a single colony was inoculated into 10 mL YPD, and also supplemented with 100 µg/mL carbenicillin (Sigma-Aldrich), and 100 µg/mL cefotaxime (TCI, Portland, OR, USA). After 24 h, cells were washed in sterile water and centrifuged at 3,220 x g for 5 min. Cultures were inoculated in triplicate to a 0.1 OD_600_ in 1 mL in a 48-well FlowerPlate with a gas-permeable sealing film cover (Excel Scientific Inc, Victorville, CA, USA) and cultivated in a Multitron incubator shaker (Infors HT, Bottmingen, Basel-Landschaft, Switzerland) at 995 rpm, 30 °C and 70% humidity. Media for this experiment was prepared by creating a defined synthetic media where total carbon was kept at 150 g/L (98 g/L glucose and 52 g/L xylose) and the nitrogen sources urea (Sigma-Aldrich) and ammonium sulfate (AS) (Sigma-Aldrich) were explored. The elemental C:N variations were 160:1, 40:1, 8:1, and 4:1 were explored, with the appropriate nitrogen source added to reach these ratios. The media composition consisted of 0.79 g/L CSM powder (Sunrise Science Products, San Diego, CA, USA), 1.7 g/L Yeast Nitrogen Base without amino acids and ammonium sulfate (Becton, Dickinson and Company,), 100 µM FeSO_4_ heptahydrate (Sigma-Aldrich), 100 mM sodium phosphate buffer pH 7.4, 100 µg/mL carbenicillin and 100 µg/mL cefotaxime. Other parameters had 40:1 AS as a base medium but excluded pH buffer, iron sulfate, antibiotics, or included 0.1% (volume/volume, or v/v) of tergitol NP-40 (Sigma-Aldrich). Cultures were also inoculated into plain YPD media, or YPD media supplemented with 2.5% (v/v) DMSO (Sigma-Aldrich) in lieu of the above defined media. Measurements were taken on days 2, 4, and 8 by taking 22 µL samples into a separate 96-well plate. Optical density and TAL measurements were taken as before, using 10% triethylene glycol (TEG) (TCI) in lieu of water for dilutions. This same procedure was followed for Fig. S1, but was set up in a BioLector and measurements were only taken on day 7 of fermentation.

For exploring TAL production from hydrolysate, a similar experimental setup was performed. TAL-producing *R. toruloides* was struck on a YPD agar plate, and a single colony was inoculated into a 50 mL culture tube with 2.5 mL YPD supplemented with 100 µg/mL carbenicillin and 100 µg/mL cefotaxime. After 24 h, 250 µL of culture was added to 995 µL YPD, 1.125 mL dry sorghum hydrolysate, and 125 µL of a nitrogen source. The nitrogen source was either pure yeast extract (Becton, Dickinson and Company), or Modified Medium 042 containing yeast extract and (MM042: 2.5 g/L corn steep, 5 g/L peptone, 2 g/L yeast extract, 1 g/L CaCl, 3 g/L CaCo3, and pH adjusted to 7.2 as defined previously [43]), and adjusted with water to provide elemental C:N ratios of approximately 40:1 and 20:1. As trace carbon and nitrogen sources might be found in hydrolysate, and the exact nitrogen composition of yeast extract varies in each batch, these elemental ratios were calculated assuming only the measured glucose and xylose amounts as the primary carbon sources, and yeast extract as the only nitrogen source (assuming yeast extract is 10% nitrogen by weight). The exact same cultures were also prepared, with an additional 31 µL DMSO. All cultures were also supplemented with 100 µg/mL carbenicillin, 100 µg/mL cefotaxime. Cells were washed in sterile water and inoculated in triplicate with a final volume of 1 mL and starting OD_600_ of 1.0 in a 48-well FlowerPlate with gas-permeable sealing film. The samples were shaken at 950 rpm, 30 °C, and 70% humidity in a Multitron. Measurements were taken on days 3 and 6 by transferring 150 µL into a 96-well plate, from which OD_600_ was measured by diluting 1 µL in 99 µL water. The remaining 148 µL was centrifuged at 4000 rpm for 5 min, the supernatant filter sterilized, and the filtrate used for TAL HPLC measurements.

### Flask cultivation conditions

A similar procedure was used to cultivate *R. toruloides* on hydrolysates in 125 mL baffled flasks. TAL-producing *R. toruloides* was struck on a YPD agar plate, and used to start a seed train by inoculating a single colony into a 50 mL culture tube with 5 mL YPD supplemented with 100 µg/mL cefotaxime and 10 µg/mL G418. After 48 h, 500 µL of culture was added to 3.25 mL YPD and 1.25 mL filtered hydrolysate of interest, supplemented with cefotaxime and G418. After 48 h, 1 mL of culture was diluted in 4 mL YPD, 5 mL filtered hydrolysate of interest, supplemented with cefotaxime and G418. Samples were grown for 48 h (or 24 h for Additional file 1: Fig. S6), and used to start experiments by diluting 2 mL cultures into 16 mL hydrolysate of interest, and 2 mL concentrated yeast extract calculated to provide ~ 20:1 C:N ratio based upon glucose and xylose estimates in the hydrolysate of interest. Solids in unfiltered hydrolysates were allowed to precipitate prior to pipetting.

At each timepoint, 500 µL of well-mixed cultures were collected from each sample. ODs were measured as previously described in filtered hydrolysates. In unfiltered hydrolysates, colony forming unit (CFU) analysis was performed to estimate growth. For this, 10 µL of well-mixed culture was diluted in 90 µL phosphate buffered saline (PBS, Sigma-Aldrich). Ten-fold serial dilutions were subsequently performed, up to 1E6 fold dilution. 10 or 20 µL of the last four volumes were plated on YPD, and the dilution that best provided distinct countable colonies was used to calculate CFUs (normalized to the volume and dilution of culture plated). For all samples, 250 µL of samples were transferred to 250 µL DMSO and vortexed for 5 min. Samples were centrifuged at max speed for 1 to 5 min, and 250 µL was filter sterilized using 0.45 μm filters for TAL HPLC analysis.

### Separations-free bioreactor fermentation

To perform bioreactor fermentation, a similar seed train (with 100 µg/mL cefotaxime and 10 µg/mL G418 at each stage) was employed but scaled up. One colony was inoculated into 25 mL YPD in a 125 mL baffled flask, grown for 48 h, and used to transfer 10 mL culture into 65 mL YPD and 25 mL filtered hydrolysate in a 500 mL baffled flask. Cultures were grown for 36 h, and used to inoculate 20 mL culture in 100 mL filtered hydrolysate and 80 mL YPD in a 1 L baffled flask. Finally, 132 mL of seed culture was used to inoculate 900.5 mL unfiltered hydrolysate supplemented with 220 mL dodecane overlay (Sigma-Aldrich) and 66 mL yeast extract calculated to give a C:N ratio of ~ 20:1 based upon glucose and xylose estimates in the hydrolysate. The culture was loaded into a 2 L Biostat B fermentor (Sartorius Stedim, Göttingen, Lower Saxony, Germany).

The batch fermentation experiment was performed in this 2 L Sartorius fermentor using unfiltered dry sorghum hydrolysate. The tank was batched with 680 mL unfiltered hydrolysate and supplemented with 40 mL of 267 g/L yeast extract solution (to a final C:N ratio of 25:1). 10% (v/v) inoculum and 20% (v/v) dodecane, containing 1 g/L pentadecane as internal standard, were added aseptically into the fermentor in the beginning of the process.

Unfiltered hydrolysate was pasteurized at 70 °C for 30 min and all the other components were filtered sterilized (0.2 μm pore size filters). To prevent bacterial contamination, cefotaxime and G418 were added to the batch medium to a final concentration of 0.1 mg/L and 0.02 mg/L, respectively.

The fermentors were controlled at 30 °C and initial pH was adjusted to pH 7 and then controlled between pH 5 and pH 8 with 2 N NaOH and 10% (v/v) H_2_SO4. The aeration was set at 0.5 vvm (volume of gas flow/volume of medium/min) and dissolved oxygen was cascade-controlled at 20% via agitation (400–1000 rpm). 10% v/v PPG-PEG-PPG antifoam (Sigma Aldrich) was used as needed to control foaming. Samples were taken in regular intervals and centrifuged to separate aqueous and solvent fractions.

At each timepoint 6 distinct 1 mL technical replicates were collected, 3 of which were used for sugars and acids analysis while the remainder were used for TAL and CFU analysis as previously described.

### Biomass feedstock

Biomass feedstocks used here are field-grown sorghum (*Sorghum bicolor*). Sorghum that was stored in the field in dry conditions (dry sorghum) was donated from Idaho National Labs (Idaho Falls, ID, USA), while sorghum that was ensiled for storage was donated from the silage pit of a commercial dairy farm in the San Joaquin Valley (Hanford, CA, USA). Dried sorghum was dried for 24 h in a 40 °C oven, while ensiled sorghum was dried under sunlight prior to transport to the laboratory. Both sources were stored at 4 °C until ready for use. Subsequently, sorghum was knife-milled with a 2 mm screen (Thomas-Wiley Model 4, Swedesboro, NJ, USA).

### One-pot biomass pretreatment and saccharification

For traditional one-pot biomass pretreatment and saccharification, pre-synthesized cholinium lysinate ([Ch][Lys]) was procured from Proionic (Grambach, Styria, Austria). For hydrolysate used in Fig. 3, sorghum, [Ch][Lys], and water (deionized, with specific resistivity of 18 MΩ·cm at 25 °C, from Purelab Flex (ELGA, Woodridge, IL, USA) were premixed in a 3:1:6 ratio (w/w) (equivalent to 30 wt% biomass loading) in a 1 L Parr 4520 series Bench Top reactor (Parr Instrument Company, model 4871, Moline, IL, USA). This pre-mixing is critical to ensure a homogeneous mixture of biomass, IL, and water. The slurry was subsequently pretreated for 3 h at 140 °C with stirring at 80 rpm powered by process (Parr Instrument Company, model: 4871) and power controllers (Parr Instrument Company, model: 4875) using three-arm, self-centering anchor with Polytetrafluoroethylene (PTFE) wiper blades. After 3 h, the pretreated slurry was cooled down to room temperature by removing the heating jacket. The pH was noted (pH ~ 10.5) and adjusted to 5.0 with concentrated hydrochloric acid (J. T. Baker, Inc., Phillipsburg, NJ, USA). Enzymatic saccharification was carried out at 50 °C for 72 h at 80 rpm using 9:1 (v/v) mixtures of cellulase (Cellic® CTec3) (Novozymes North America, Franklinton, NC, USA) and hemicellulase (HTec3 NS 22244) (Novozymes) at a loading of 10 mg protein per 1 g biomass. After 72 h, hydrolysate was separated from the residual solids by centrifugation followed by the filtration of the supernatant through 0.45 μm sterile filter units. The pH of the filtered hydrolysate was adjusted to 7.0 using an aqueous solution of 2 M sodium hydroxide (Sigma-Aldrich), and subsequently filtered through 0.2 μm sterile filter units.

For Fig. 4 and Additional file 1: Figures S4, S5 and S6, the same steps were performed with the following modifications. A sorghum:[Ch][Lys]:water mixture using a 2:1:7 (equivalent to 20 wt% biomass loading) was premixed in a 10 L Hastelloy C276 Parr vessel (Parr Instrument company, model: 4555-58). Reaction temperature was increased to 160 °C, and speed reduced to 50 rpm. pH was subsequently adjusted to 5.0 using 50% (w/w) H_2_SO_4_ for saccharification, where speed was again reduced to 50 rpm and enzyme loading increased to 30 mg/g biomass. pH was adjusted back to 7.0 prior to filter sterilizing. For a subset of these hydrolysates, samples were not filter sterilized and instead adjusted to a pH of 7.0 and pasteurized at 80 ℃ for 1 h prior to fermentation.

For Figs. 5 and 6, IL was prepared in-situ in the one-pot reaction in lieu of the IL being pre-synthesized in a separate reaction. For this, cholinium hydroxide ([Ch][OH]) (Proionic) was used in lieu of [Ch][Lys], again using a sorghum:[Ch][OH]:water ratio of 2:1:7 for both dried and ensiled sorghum. An amount of lactic acid and acetic acid equivalent to the amounts estimated in ensiled biomass were added to the dried biomass to facilitate in-situ IL synthesis.

### Analytical methods

TAL was quantified using a phenolic compound analysis similar to previously described methods [19, 62]. Briefly, an Eclipse Plus Phenyl-Hexyl column (250 mm length, 2.6 mm diameter, 5 μm particle size) (Agilent Technologies) was loaded onto an Agilent Technologies 1260 infinity series high-performance liquid chromatography (HPLC) system equipped with a Refractive Index Detector. Two aqueous mobile phases consisting of water supplemented with 10 mM ammonium acetate and 0.7% formic acid (Solvent A) and 90% acetonitrile supplemented with 10 mM ammonium acetate and 0.7% formic acid (Solvent B) were used, with a profile mixture as follows: 30% Solvent B, 0.5 mL/min for 12 min, 80% Solvent B, 0.5 mL/min for 0.1 min, 100% Solvent B, 0.5 mL/min for 0.5 min, 100% Solvent B, 1.0 mL/min for 0.2 min, and 30% Solvent B, 1.0 mL/min for 2.8 min. TAL concentrations were compared against analytical standards using 254, 280, and 310 nm spectral profiles. Column temperature was kept at 50 °C.

Sugars (glucose and xylose) and organic acids (lactic acid and acetic acid) were quantified on an Agilent HPLC 1260 infinity system (Agilent Technologies) equipped with an Aminex™ HPX-87 H column (Bio-Rad, Hercules, CA, USA) and a Refractive Index detector. An aqueous solution of sulfuric acid (4 mM) was used as the eluent (0.6 mL/min, column temperature 60 °C). Standards for quantification were obtained from Sigma-Aldrich.

### Sequencing of bacterial contamination

Contaminating bacteria observed in bioreactor fermentations were recovered from colonies formed on YPD plates during CFU analysis, inoculated into 5 mL YPD, and grown overnight at 30 °C with 200 rpm shaking. A colony PCR was performed to amplify DNA encoding 16 S rRNA using primers 8 F (AGAGTTTGATCCTGGCTCAG) and U1492R (GGTTACCTTGTTACGACTT) as previously outlined [63]. The resulting DNA was sanger sequenced (Azenta Life Sciences, South San Francisco, CA, USA) and NCBI blast was used to search for the genus. The recovered sequence shared 99.4% homology with *Lysinibacillus macroides*.

## Supplementary Information

**Additional file 1:Figure S1**. The experiment presented in Fig. 2 was repeated, and reproducible titers were obtained at a maximum of a C:N ratio of 40:1 for both urea and ammonium sulfate media, with a dip in TAL titers at a C:N of 8:1 in urea media. Error bars represent standard deviation of biological triplicates.**Figure S2**. Addition of buffer slightly improves TAL production. All buffers were added at a concentration of 100mM. Error bars represent standard deviation of biological triplicates.**Figure S3.** Antibiotic addition slows early growth on day 1 in YPD, but does not reduce maximum titers later on. TAL was measured after four days of growth. Antibiotic concentrations are listed in working concentration (µg/mL) under each condition in the top graph, which reflects the identical order in the bottom graph. Error bars represent standard deviation of biological triplicates.**Figure S4.** Comparison of hydrolysates derived from field-grown sorghum biomass that was stored via ensilage, or was dried prior to storage. Fermentation was performed in bench-scale flask cultures. (**A**) Titers obtained during fermentation over time. (**B**) Glucose during fermentation. (**C**) Xylose during fermentation. (**D**) Total viable colony forming units (CFUs) obtained at the end of the experiment. All error bars represent standard deviations of biological triplicates.**Figure S5.** The effect of addition of 1.25% DMSO to enhance TAL titers from ensiled hydrolysates produced from the same batch as in Supplementary Figure S4. Error bars represent standard deviation of biological triplicates.**Figure S6.** The same experiment performed in parallel to Fig. 4, but using only one day of pre-adaption in the final step of the seed culturing. In this case, a slight delay in growth in both dry and ensiled hydrolysates occurred during the first day. (**A**) Titers obtained during fermentation on filtered hydrolysates over time. (**B**) Growth in filtered hydrolysates, as measured by optical densities. (**C**) Primary carbon sources during fermentation on filtered hydrolysates, at the experiment start and end. (**D**) Titers obtained during fermentation on unfiltered, pasteurized hydrolysates over time. (**E**) Growth in unfiltered hydrolysates, as measured by viable colony forming units (CFUs) of *R. toruloides* on YPD plates. The limit of detectable colony units is indicated (1000 units/mL). (**F**) Primary carbon sources during fermentation on unfiltered hydrolysates, at the experiment start and end. All error bars represent standard deviations of biological triplicates.**Figure S7.** Analysis of partition coefficient of TAL between water and various common solvents used as overlays in bioreactor fermentations. Water was mixed at a 1:1 ratio with each solvent, and 6 g/L TAL was added. Subsequently, the aqueous phase was analyzed for TAL titers in five technical replicates. Error bars represent standard deviation. The partition coefficient between dodecane, durasyn164, and oleyl alcohol and water were calculated as 1.11, 0.59, and 2.68 respectively.
