# Supplementary material for: Advanced one-pot deconstruction and valorization of lignocellulosic biomass into triacetic acid lactone using Rhodosporidium toruloides
Source: Microb Cell Fact. 2022 Dec 8;21:254. doi: 10.1186/s12934-022-01977-0 (PMC9733078; doi:10.1186/s12934-022-01977-0)
Supplement: Supplementary file 1 — Additional file 1:Figure S1. The experiment presented in Fig. 2 was repeated, and reproducible titers were obtained at a maximum of a C:N ratio of 40:1 for both urea and ammonium sulfate media, with a dip in TAL titers at a C:N of 8:1 in urea media. Error bars represent standard deviation of biological triplicates.Figure S2. Addition of buffer slightly improves TAL production. All buffers were added at a concentration of 100mM. Error bars represent standard deviation of biological triplicates.Figure S3. Antibiotic addition slows early growth on day 1 in YPD, but does not reduce maximum titers later on. TAL was measured after four days of growth. Antibiotic concentrations are listed in working concentration (µg/mL) under each condition in the top graph, which reflects the identical order in the bottom graph. Error bars represent standard deviation of biological triplicates.Figure S4. Comparison of hydrolysates derived from field-grown sorghum biomass that was stored via ensilage, or was dried prior to storage. Fermentation was performed in bench-scale flask cultures. (A) Titers obtained during fermentation over time. (B) Glucose during fermentation. (C) Xylose during fermentation. (D) Total viable colony forming units (CFUs) obtained at the end of the experiment. All error bars represent standard deviations of biological triplicates.Figure S5. The effect of addition of 1.25% DMSO to enhance TAL titers from ensiled hydrolysates produced from the same batch as in Supplementary Figure S4. Error bars represent standard deviation of biological triplicates.Figure S6. The same experiment performed in parallel to Fig. 4, but using only one day of pre-adaption in the final step of the seed culturing. In this case, a slight delay in growth in both dry and ensiled hydrolysates occurred during the first day. (A) Titers obtained during fermentation on filtered hydrolysates over time. (B) Growth in filtered hydrolysates, as measured by optical densities. (C) Primary carbon sources [file 12934_2022_1977_MOESM1_ESM.docx]

# **Additional Information to:**

# **Advanced one-pot deconstruction and valorization of lignocellulosic biomass into triacetic acid lactone using *Rhodosporidium toruloides***

**Peter B. Otoupal, Gina M. Geiselman, Asun M Oka, Carolina A. Barcelos, Hemant Choudhary, Duy Dinh, Wenqing Zhong, HeeJin Hwang, Jay D. Keasling, Aindrila Mukhopadhyay, Eric Sundstrom, Robert W. Haushalter, Ning Sun, Blake A. Simmons, & John M. Gladden**

#


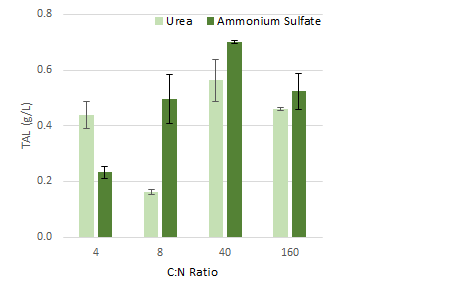


**Figure S1** - The experiment presented in Figure 2 was repeated, and reproducible titers were obtained at a maximum of a C:N ratio of 40:1 for both urea and ammonium sulfate media, with a dip in TAL titers at a C:N of 8:1 in urea media. Error bars represent standard deviation of biological triplicates.


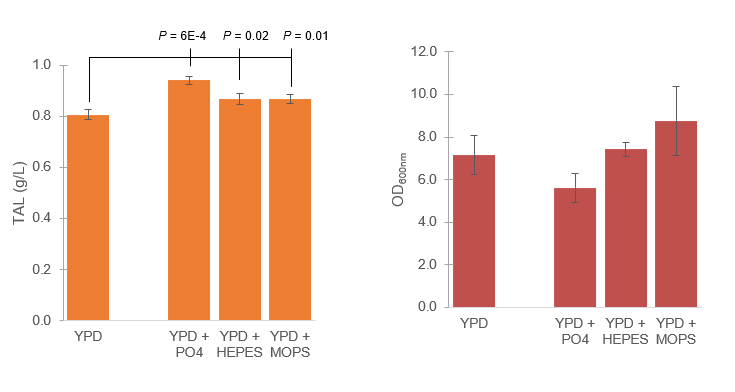


**Figure S2** - Addition of buffer slightly improves TAL production. All buffers were added at a concentration of 100mM. Error bars represent standard deviation of biological triplicates.


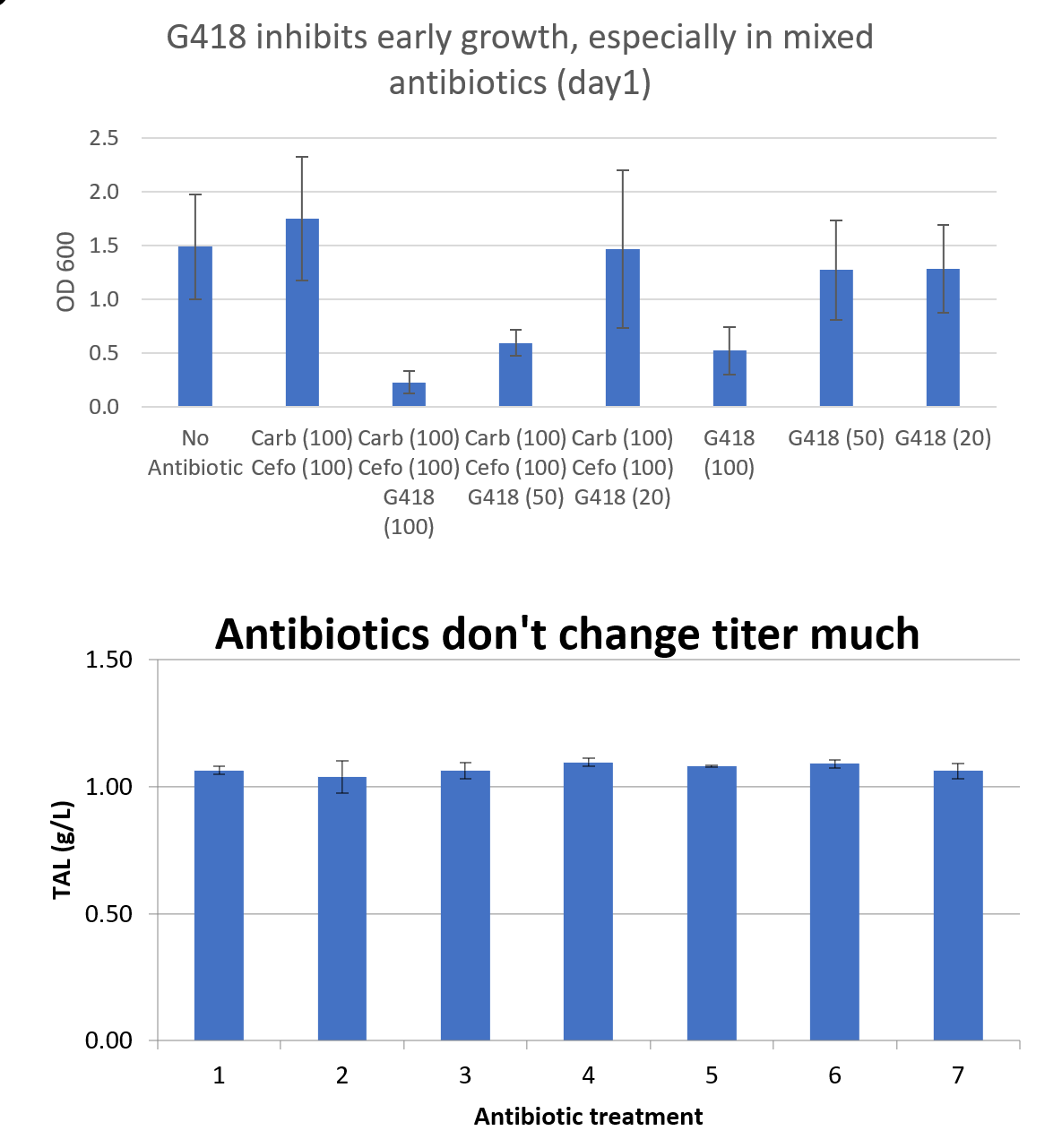


**Figure S3** - Antibiotic addition slows early growth on day 1 in YPD, but does not reduce maximum titers later on. TAL was measured after four days of growth. Antibiotic concentrations are listed in working concentration (µg/mL) under each condition in the top graph, which reflects the identical order in the bottom graph. Error bars represent standard deviation of biological triplicates.


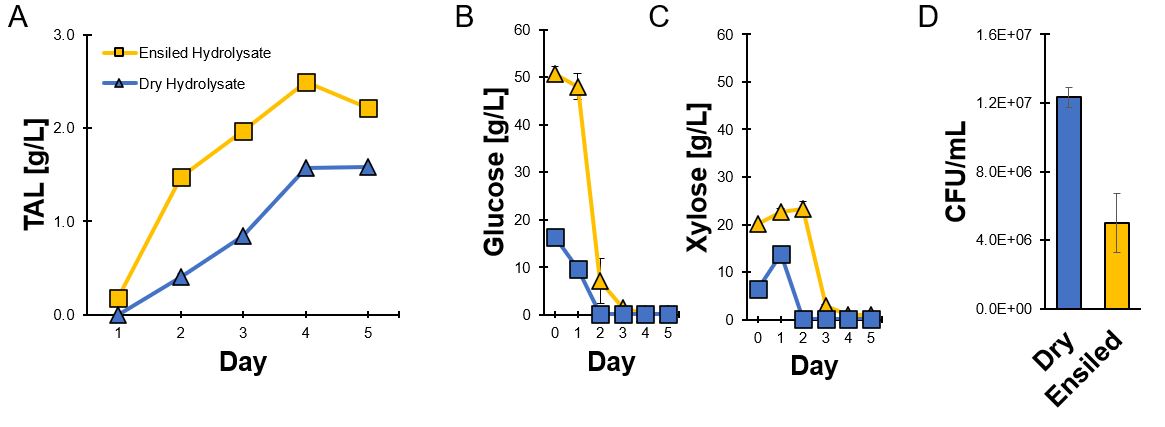


**Figure S4.** Comparison of hydrolysates derived from field-grown sorghum biomass that was stored via ensilage, or was dried prior to storage. Fermentation was performed in bench-scale flask cultures. (**A**) Titers obtained during fermentation over time. (**B**) Glucose during fermentation. (**C**) Xylose during fermentation. (**D**) Total viable colony forming units (CFUs) obtained at the end of the experiment. All error bars represent standard deviations of biological triplicates.


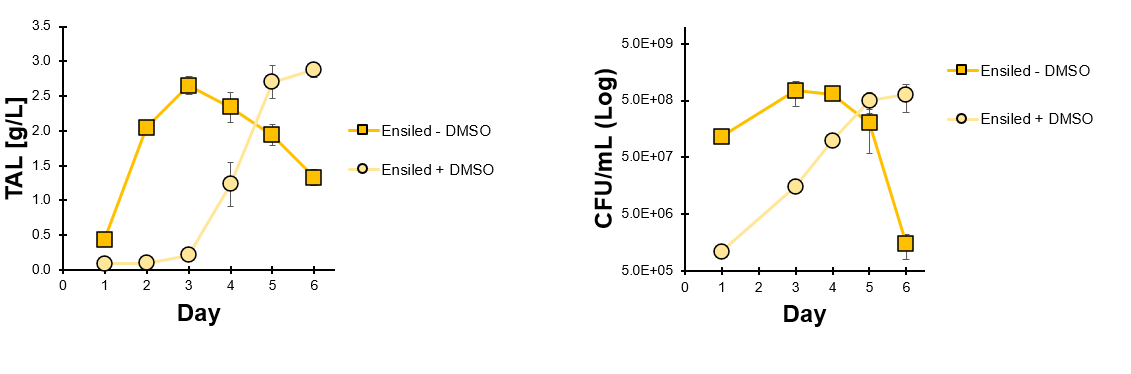


**Figure S5.** The effect of addition of 1.25% DMSO to enhance TAL titers from ensiled hydrolysates produced from the same batch as in Supplementary Figure S4. Error bars represent standard deviation of biological triplicates


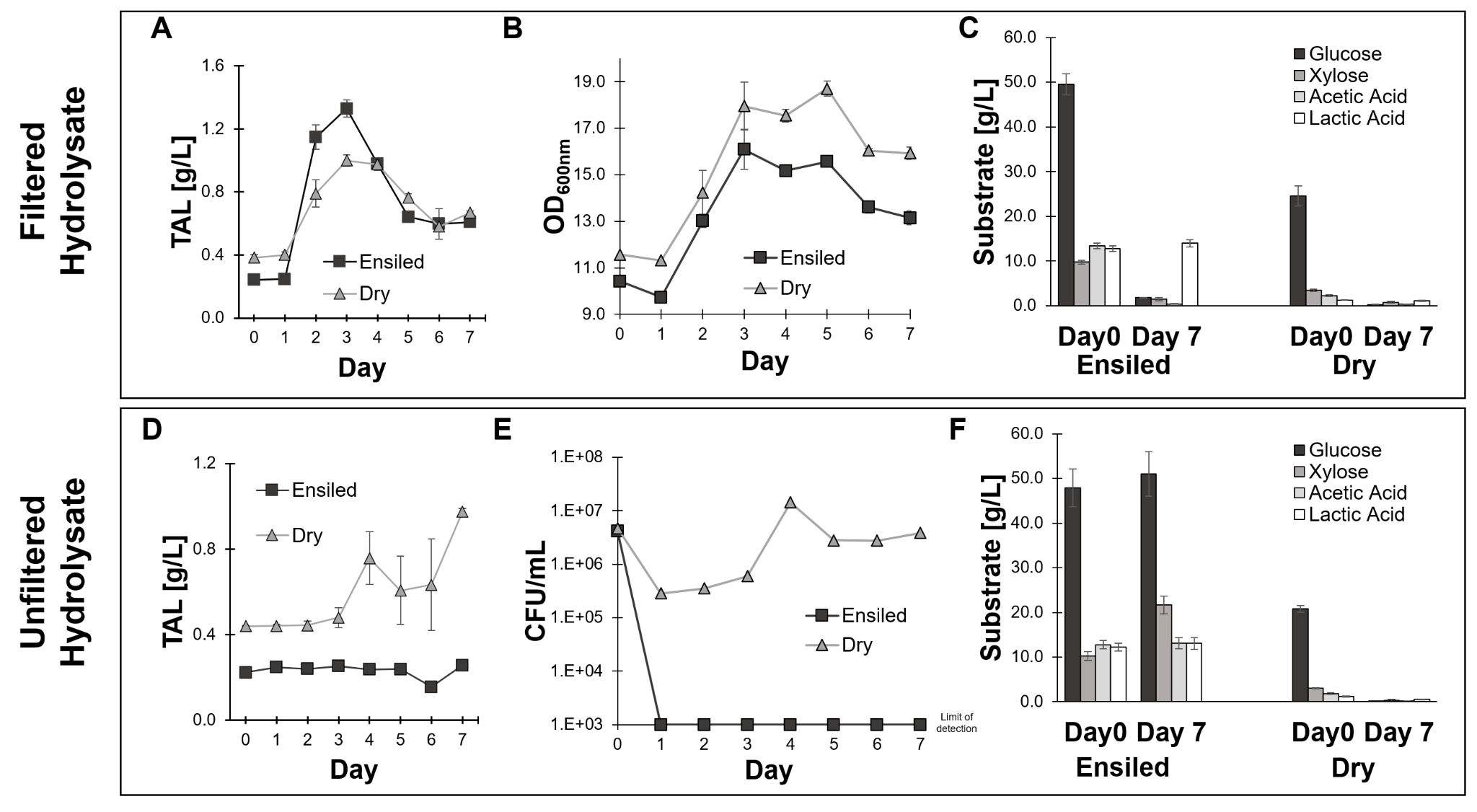


**Figure S6.** The same experiment performed in parallel to Figure 4, but using only one day of pre-adaption in the final step of the seed culturing. In this case, a slight delay in growth in both dry and ensiled hydrolysates occurred during the first day. (**A**) Titers obtained during fermentation on filtered hydrolysates over time. (**B**) Growth in filtered hydrolysates, as measured by optical densities. (**C**) Primary carbon sources during fermentation on filtered hydrolysates, at the experiment start and end. (**D**) Titers obtained during fermentation on unfiltered, pasteurized hydrolysates over time. (**E**) Growth in unfiltered hydrolysates, as measured by viable colony forming units (CFUs) of *R. toruloides* on YPD plates. The limit of detectable colony units is indicated (1000 units/mL). (**F**) Primary carbon sources during fermentation on unfiltered hydrolysates, at the experiment start and end. All error bars represent standard deviations of biological triplicates.

**
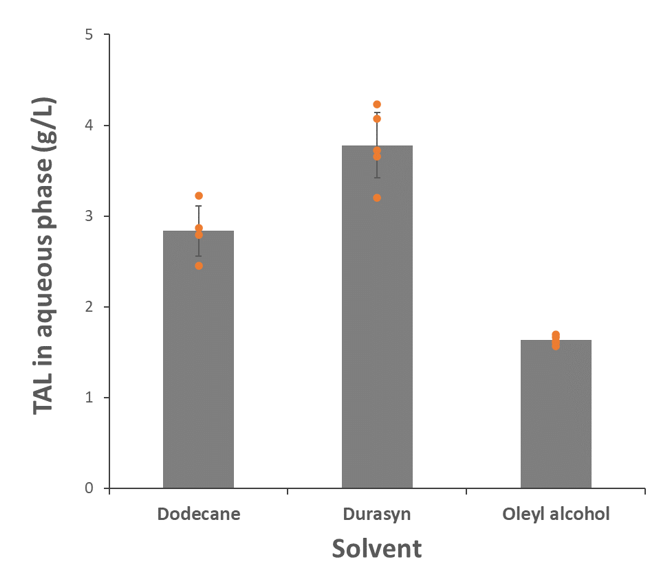
**

**Figure S7.** Analysis of partition coefficient of TAL between water and various common solvents used as overlays in bioreactor fermentations. Water was mixed at a 1:1 ratio with each solvent, and 6 g/L TAL was added. Subsequently, the aqueous phase was analyzed for TAL titers in five technical replicates. Error bars represent standard deviation. The partition coefficient between dodecane, durasyn164, and oleyl alcohol and water were calculated as 1.11, 0.59, and 2.68 respectively.
